# An Eco-Friendly Synthetic Approach for Copper Nanoclusters and Their Potential in Lead Ions Sensing and Biological Applications

**DOI:** 10.3390/bios12040197

**Published:** 2022-03-26

**Authors:** Sayed M. Saleh, Wael A. El-Sayed, May A. El-Manawaty, Malek Gassoumi, Reham Ali

**Affiliations:** 1 Department of Chemistry, College of Science, Qassim University, Buraidah 51452, Saudi Arabia; w.shendy@qu.edu.sa; 2 Chemistry Branch, Department of Science and Mathematics, Faculty of Petroleum and Mining Engineering, Suez University, Suez 43721, Egypt; 3 Photochemistry Department, National Research Centre, Dokki, Giza 12622, Egypt; 4 Pharmacognosy Department, Pharmaceutical and Drug Industries Research Institute, National Research Centre, 33 El Buhouth Street, Cairo 12622, Egypt; mayalyem@gmail.com; 5 Department of Physics, College of Science, Qassim University, P.O. Box 64, Buraidah 51452, Saudi Arabia; malek.gassoumi@gmail.com; 6 Laboratory of Condensed Matter and Nanosciences, University of Monastir, Monastir 5000, Tunisia; 7 Chemistry Department, Science College, Suez University, Suez 43518, Egypt

**Keywords:** copper nanoclusters, eco-friendly synthesis, pepsin, optical sensing, metal ions, anticancer

## Abstract

A new preparation route for high-luminescent blue-emission pepsin copper nanoclusters (Pep-CuNCs) is introduced in this work. The synthesized nanoclusters are based on a pepsin molecule, which is a stomach enzyme that works to digest proteins that exist in undigested food. Here, we have developed an eco-friendly technique through microwave-assisted fast synthesis. The resulting copper nanoclusters (CuNCs) exhibit significant selectivity towards Pb(II) ions. The pepsin molecule was utilized as a stabilizer and reducing agent in the production procedure of Pep-CuNCs. The characteristics of the resulting Pep-CuNCs were studied in terms of size, surface modification, and composition using various sophisticated techniques. The CuNCs responded to Pb(II) ions through the fluorescence quenching mechanism of the CuNCs’ fluorescence. Thus, great selectivity of Pep-CuNCs towards Pb(II) ions was observed, allowing sensitive determination of this metal ion at lab-scale and in the environment. The CuNCs have detection limits for Pb(II) in very tenuous concentration at a nanomalar scale (11.54 nM). The resulting Pep-CuNCs were utilized significantly to detect Pb(II) ions in environmental samples. Additionally, the activity of Pep-CuNCs on different human tumor cell lines was investigated. The data for the observed behavior indicate that the Pep-CuNCs displayed their activity against cancer cells in a dose dependent manner against most utilized cancer cell lines.

## 1. Introduction

With significant industrial progress in many fields, the need for the determination of lead has increased. Lead ions are present and dispersed in varying amounts in nature, as they are present in many ores such as galena, anglesite, and cerussite. Lead is used in many industries, including for storage batteries, cable sheaths, weaponry, and radioactive shielding. Lead is used in large quantities and added to gasoline to improve its properties. Environmental lead pollution has caused many problems, and many countries have made arrangements to reduce the use of lead in gasoline. However, lead in the environment is recycled to be reused for valuable purposes. In general, lead compounds emitted into the air typically end up in water sources [1]. Lead can destroy the nervous system and cause kidney failure, and is recognized as a carcinogen [2]. Lead is generally found in the environment in the form of inorganic lead(II) compounds. The Environment Protection Agency (EPA) and World Health Organization (WHO) stated that acceptable concentrations of lead ions in water are 15 ppb and 10 ppb [3]. Various instruments have been utilized to detect lead ions in water. These techniques include atomic absorption spectrometry, and inductively coupled mass-plasma spectrometry. The difficulties with these techniques are that they are expensive, time-consuming, and need a well-equipped laboratory. However, label-free methods based on metal nanoclusters have distinctive optical properties and tailorable surface functionalization that can detect different types of analytes [4].

Luminescent nanoscale particles have obtained much attention due to their distinctive characteristics in various applications, including biotechnology, medicine, cell imaging, and labeling [5,6,7]. Recently, different types of luminescent nanoparticles have been detected such as luminescent protein [8,9], medicinal probe [10], natural chromophore [11,12], quantum dots [13], carbon dots [14], metal-organic framework [15], fluorescent inorganic-organic hybrid nanoparticles [16] and noble metal nanoclusters [17]. These fluorescent nanomaterials have many significant applications in sensors, molecular imaging, and nanomedicine because of their outstanding optical characteristics, the extreme specific surface-to-volume ratio, superficial surface modification, and the facility of being tenable [18,19].

Nowadays, with significant development in all areas of technology, noble metal nanoclusters have an essential role in developing biological and chemical sensors [20,21]. Furthermore, they can be used to produce high-precision devices to enhance biological sensing and provide more sensitive and accurate biomolecular diagnostics. The nano-size of the particles characterizes unique chemical and physical properties and has led to the development of a group of biosensors. The applications of these sensors include: (a) diagnosing some crucial diseases [22], (b) tracking and detecting different microbes [23,24], (c) imaging living cells [25], (d) studying numerous reactions inside living cells [26], (e) tracking disease symptoms or monitoring drug treatment and other applications [27] that may benefit humans revealing information about biological systems for scientific research. In general, the participation of metallic nanoclusters has contributed significantly to the development of bio-nanosensors due to the ease of application and the large surface area of the particles. The metal nanoclusters, which have precious photophysical properties, have various advantages including considerable photostability, biocompatibility, small size, ease of synthesis, eco-friendliness, and large Stokes shift. The nanoclusters’ composition includes a crystal structure consisting of a few atoms that may reach 25 atoms in some combinations. It, therefore, has a size of less than 2 nanometers [28].

**Scheme 1:**
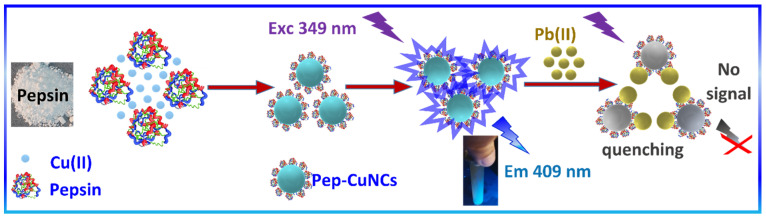
Synthesis scheme of Pep-CuNCs and their application in Pb(II) ions sensing.

Herein, we used pepsin enzyme as a significant ligand and capping/reducing agent for the preparation of fluorescent Pep-CuNCs (Scheme 1). Pepsin is a chain protein exhibiting a catalytic site that is formed at the junction of the domain, each domain contains two aspartic acid residues. Thus, pepsin has free carboxylic and amino groups that can react as a polyvalent ligand to reduce Cu(II) ions and stabilize the metal core, leading to the formation of stable fluorescent CuNCs. The prepared Pep-CuNCs were utilized for sensing Pb(II) metal ions. We believe that the synthesized Pep-CuNCs are promising materials for biological applications.

## 2. Materials and Methods

### 2.1. Materials

The pepsin, lead nitrate, ethylenediaminetetraacetic acid (EDTA) and all chemicals and reagents were purchased from Sigma Aldrich Co. (Taufkirchen, Germany) (http://www.sigmaaldrich.com, accessed on 25 February 2022), at the maximum purity offered. Dialysis membrane of 12–14 KD was obtained from the Spectrum chemical Co. (New Brunswick, NJ, USA) (https://www.spectrumchemical.com/chemicals/Dialysis-Membrane, accessed on 25 February 2022). The organic solvents were of the best grade available.

### 2.2. Apparatus and Instrumentation

The absorption spectra of the studied solutions were observed in a 1 cm quartz cell using an Evolution™ 200 series (Thermo Fisher Scientific, Waltham, MA, USA) UV-Vis spectrophotometer. Both emission and excitation spectra were collected with a JASCO-FP8200 spectrofluorometer using 1 cm quartz for all spectral measurements. High resolution transmission electron microscope (HR-TEM) images of nanoclusters were attained utilizing JEOL-100S Japan functioning at 200 kV. Fourier Transform Infrared Spectroscopy (FTIR) spectra were recorded on a 4100 Jasco-Japan (4000 to 400 cm^−1^). Finally, X-ray Photoelectron Spectroscopy (XPS) analyses were collected on a K-ALPHA (Thermo Fisher Scientific, USA) with monochromatic X-ray Al K-alpha radiation −10 to 1350 eV spot size 400 µm at pressure 10^−9^ mbar with full-spectrum pass energy 200 eV and narrow-spectrum 50 eV.

### 2.3. Synthesis of Pep-CuNCs

All glass was cleaned carefully in aqua regia and then washed twice with bi-distilled water. Pep-CuNCs were prepared in the aqueous medium according to the following procedure. Here, the pepsin molecules were involved in this method as a stabilizing and reducing agent. Into a 25 mL round flask and at 37 °C, 4 mL of 10 mM Cu(NO_3_)_2_ was added to 6 mL of 20 mg/mL Pepsin aqueous solution. The resulting solution was stirred vigorously for 3 min, then 300 µM NaOH ml of 1M NaOH was added at once, and the resulting mixture was stirred for another 3 min. The solution was transferred to a 10 mL microwave tube. The reaction mixture was kept in the microwave oven (850 W, Anton paar monowave 200) with irradiation power for 30 min. After the completion of the irradiation, the produced microwave-assisted solution was dialyzed in ultra-pure water overnight to remove all contaminants. After dialysis, Pep-CuNCs were stored at 4 °C in the dark. The Quantum Yield (QY) of Pep-CuNCs was calculated by quantifying the integrated fluorescence intensities of the Pep-CuNCs and the reference compound quinine sulfate (QY of 55%) in 0.05 M H_2_SO_4_ under 310 nm excitation [29].

### 2.4. Detection of Metal Ions

The stock solution of Pep-CuNCs aqueous solution was prepared by diluting the as-prepared nanocluster solution 10-fold using an appropriate amount of Milli-Q water. We used the resulting solutions to take the subsequent measurements. Then, solutions including the Pep-CuNCs and 0.1 mM stock solutions of the studied metal ions were prepared and the fluorescence measurements were taken under excitation of 349 nm. Different ions involving alkali, alkaline, and transition metals were tested utilizing the same conditions for further study of selectivity and sensitivity.

### 2.5. Biological Application—In-Vitro Methods

#### 2.5.1. Human Tumor Cell Lines Utilized

A431 epidermoid carcinoma, HCT-116 colorectal carcinoma, PC3 prostate adenocarcinoma, A-549 lung adenocarcinoma, MCF-7 breast cancer adenocarcinoma, MIA PaCa2 pancreatic carcinoma and HOS osteocarcinoma cells were kindly supplied by Prof. Dr. Stig Linder, Karolinska institute, Sweden.

#### 2.5.2. Monolayer Cytotoxicity Bioassay

To maintain the cells’ viability, they were sub-cultured twice a week using DMEM-F12 medium supplemented with l-glutamine and fetal bovine serum added at a concentration of 5–15%, and the flasks kept at 37 °C incubation under 5% CO_2_ and 95% humidity. Trypsin versine at 0.15 % was used for detaching the cells. When preparing the working 96-well-plates, 10,000 to 20,000 cells per well were seeded, but in case of normal cells 60,000 cells per well were prepared, and after a day the cells reached a concentration of about 80%. Under those conditions the test samples were added in triplicate at 100 µM, 50 µM, 25 µM and 12.5 µM and the plates were left incubated for 72 h. Positive (Doxorubicin) and negative controls were prepared with the test samples [30,31,32]. MTT [3-(4,5-dimethylthiazol-2-yl)-2,5-diphenyltetrazolium bromide] salt was used to measure cell viability [3]. The resulting blue color was measured at 595 nm with reference 690 nm. Percentage viability was calculated by dividing the absorbance of the test well by the negative control, and multiplying by 100. If the cytotoxicity percentage is required, we can simply subtract the percentage viability from 100.

#### 2.5.3. IC_50_ Determination

The samples which were shown to be active on the cells were tested. Graphpad PRISM (6.01) was used to analyze the results using the nonlinear regression method, by which we obtained the IC_50_ values.

## 3. Results and Discussion

### 3.1. Optical Characteristics

**Figure 1:**
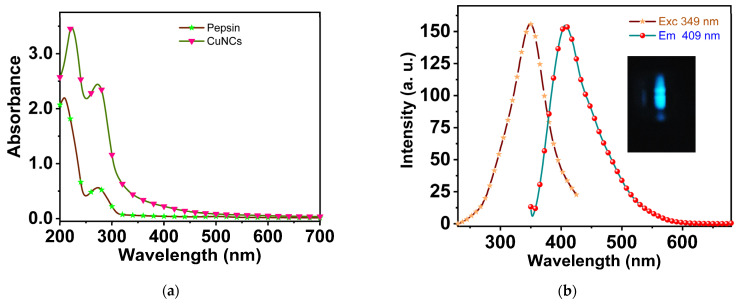
(**a**) The absorption spectra of pepsin and Pep-CuNCs; (**b**) The excitation and emission spectra of Pep-CuNCs.

The UV-vis absorbance of the synthesized Pep-CuNCs solution did not show the production of nanoparticles with core diameters large than 2 nm, because the absence of delocalized surface plasmon resonance (SPR) peaked at around ~600 nm. The blue shift of the absorption peak of Pep-CuNCs is consistent with the observation that the CuNCs were smaller in size than 2 nm [33]. The wavelength of luminescent CuNCs was based on the size of the nanoclusters’ cores. Figure 1 displays the emission spectra of Pep-CuNCs. The blue emission peak of Cu NCs was detected at (λ_em_) of 409 nm under the excitation wavelength of (λ_ex_) of 360 nm. The quantum yield for the as-prepared Pep-CuNCs was found to be 17%.

### 3.2. Particles’ Characterizations

**Figure 2:**
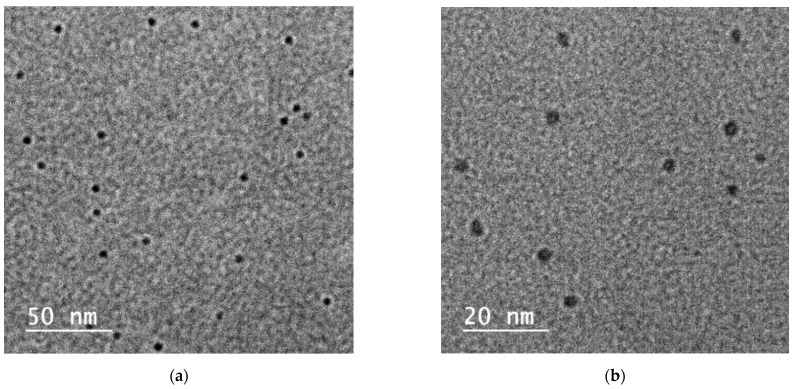

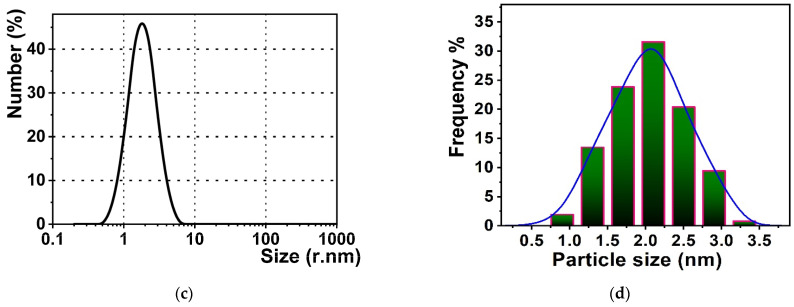
(**a**,**b**) TEM images, (**c**) DLS, and (**d**) SD histogram of the as-prepared Pep-CuNCs.

TEM, DLS, and SD analyses were carried out to detect the typical size and surface morphology of the as-prepared Pep-CuNCs. Figure 2a,b shows the TEM data of the CuNCs, indicating that the synthesized nanoclusters were significantly well-dispersed and had uniform shapes with a similar diameter of approximately ~2 nm. Therefore, the obtained findings confirm that the pepsin reduction procedure is a usefultechnique for synthesizing copper nanoclusters, giving excellent results.. The DLS and size distribution (SD) histograms (Figure 2b,d) reveal that the Pep-Cu-NCs formed uniform well-dispersed clusters in the aqueous solution. The results were confirmed by the TEM measurements and the main particle size was assessed to be ~2 nm.

**Figure 3:**
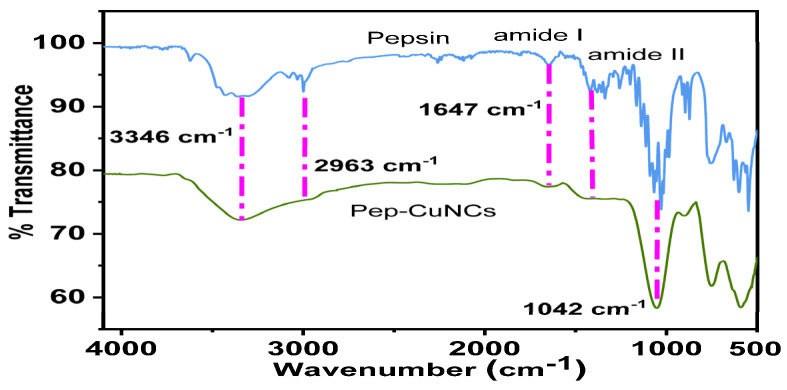
(blue) FTIR spectra of pepsin. (green) Pep-CuNCs.

To detect the active chemical groups that exist in the Pep-CuNCs, IR spectra were carried out. Figure 3 exhibits FTIR data of pure pepsin (blue) and the Pep-CuNCs (green), respectively. The broad peak at 3300 and 2930 cm^−1^ coreresponds with the hydroxy and C−H groups stretching on the surface of the nanoclusters and the natural protein. Moreover, pepsin provides two main bands at 1642 and 1406 cm^−1^, resulting in the amide I (C=O stretching) and amide II (CH_3_ asymmetric bending) peaks of the protein. On the other hand, the IR spectrum of surface-modified nanoclusters indicated that the strength of the amide II peak was weakened in Pep-CuNCs. Also, the amide I band disappeared, which may be attributed to the chelation of Cu atoms on CuNCs to pepsin. Additionally, the differences between the spectra of pepsin and of the as-prepared nanoclusters prove the chelation process was established through the present active sites of pepsin, such as mercapto, carboxyl, and amino groups.

**Figure 4:**
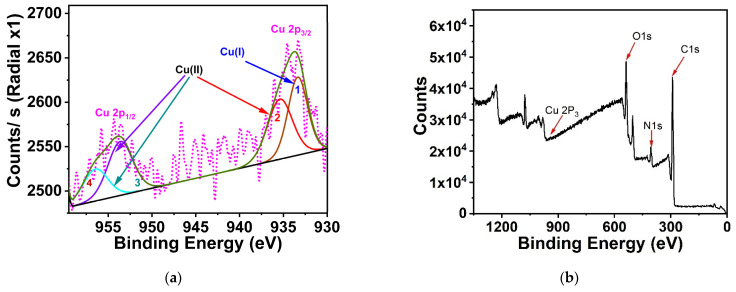
(**a**) XPS spectra of Cu2P for Pep-CuNCs; (**b**) The survey XPS spectrum of Pep-CuNCs.

To further characterize the electronic structures of the copper element in the nanoclusters, XPS analyses were collected (Figure 4a). The Cu XPS 2p core level spectrum had two sets of 2p bands; one set had Cu 2p_3/2_ and the other Cu 2p_1/2_. These bands localize at 930.7 and 950.6 eV correspondingly, indicating the presence of copper with low valance, consisting of Cu and Cu(I) (Figure 4a) [34]. Additionally, the distinct peaks of Cu 2p_3/2_ at 931.77 eV and Cu 2p_1/2_ at 953.61 eV in combination with the main bands at 956.39 and 935.34 eV are typical characteristics of Cu(II), initiating from the predictable oxidation of Cu/Cu(I) metal/[oxidant] in these nanoclusters. The XPs survey spectra shown in Figure 4b reveals the existence of Cu, C, N, and O in the yield.

### 3.3. Sensing Process

**Figure 5:**
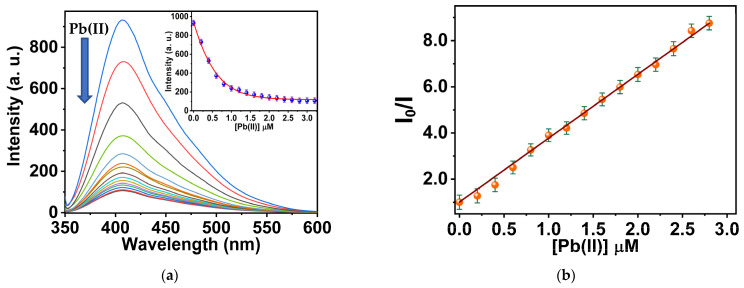
(**a**) Fluorescent emission of Pep-CuNCs in the presence of various concentration of Pb(II) ions from 0.1 to 6 mM; (**b**) Classical Stern-Volmer plot of fluorescence intensities ratio to the Pb(II).

The quenching process of the Pep-CuNCs on diverse Pb(II) concentrations was also studied. Figure 5a shows that the quenching influences happened instantly when Pb(II) was added to the Pep-CuNCs aqueous solution, and the increment of quenching consequence attained a constant value in less than 10 s. Such rapid response is of distinct significance in different applications where particular samples can be examined immediately. Without Pb(II), Pep-CuNCs exhibited no considerable alteration in emission intensity during the investigation test, which proved that the quenching influence was really due to the addition of Pb(II). The as-synthesized Pep-CuNCs were remarkably unchanged under the optimum synthesis conditions. The stability of CuNCs based on their fluorescence intensity was examined and there was no significant decrease in the nanoclusters’ emission intensity at room temperature for 12 h, enabling them to be adducted for several applications. Notably, given the rapid turn-off process of Pep-CuNCs in the presence of Pb(II), it is conceivable that a novel Pb(II) optical chemosensor may be acquired that can be applied to other instruments that need several complicated preliminary steps for sample preparations.

The sensitivity of the Pep-CuNCs-Pb(II) chemical system was examined by changing the concentration of Pb(II) ions. Figure 5a displays the sensitivity of emission intensity to the rising concentration of Pb(II), and its proportional reduction with no obvious change to the peak maxima or peak shape of fluorescence spectra accompanying the emission quenching. Furthermore, during the titrimetric reaction Pep-CuNCs-Pb(II), when Pb(II) concentration reached 3 µM, the emission intensity was quenched, and no additional decrease took place. Figure 5b shows the relation of the luminescence intensity of Pep-CuNCs at 409 nm to Pb(II) concentration. The dynamic range of this significant reaction was between LOQ (10/3 LOD) ~35 nM and 3 µM with square root R^2^ 0.99923, which can be illustrated by the classical Stern–Volmer equation, where I_0_/I = 1.0 + K_sv_[Q] where I_0_ and I are the emission intensity of Pep-CuNCs in the absence and presence of Pb(II), respectively, and K_sv_ is the Stern–Volmer quenching constant. The K_sv_ value was calculated to be 2.7 × 10^6^ M^−1^ based on the slope of the Stern–Volmer equation. Also, the LOD of this system was estimated to be 11.54 nM, supposing that the luminescence intensities were detected with a precision of ±1% [35]. The value of the LOD is considerably below the limit value of Pb (II) in drinking water (72 nM)stated by the United States Environmental Protection Agency (EPA) [36]. Herein, this novel chemosensor delivered a significant LOD value which is a considerable improvement on other applied fluorescence-determination techniques [37,38].

On consecutive additive of Pb(II) cations to the Pep-CuNCs, we noticed the fluorescence turn-off mechanism reach about 21.66% quenching regarding the existence of 0.25 µM Pb(II) ions. On increased addition of Pb(II) to the Pep-CuNCs aqueous solution, the fluorescence intensity was 88.16% for ~3 µM Pb(II) concentration (Figure 5). The Pep-CuNCs-Pb(II) “turn-off” mechanism to be proposed is as follows: the surface of the pepsin-stabilized CuNCs was encased by Pb(II) with the existence of pepsin moiety as a reducing and stabilizing agent. The Pb(II) ions interacted with Cu(I) at the surface of the copper nanocluster with significant electronic structure 5d^10^-Pb(II) and 3d^10^-Cu(I) metallophilic reaction to stimulate quenching of the CuNCs’ fluorescence [39,40].

**Figure 6:**
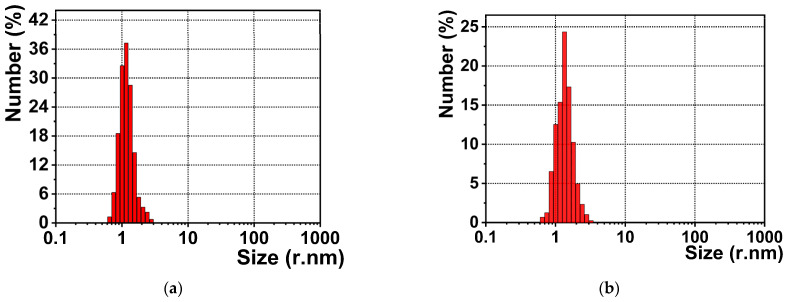

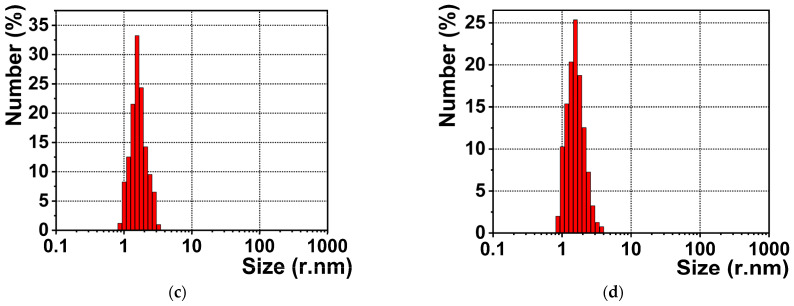
DLS data of Pep-CuNCs in the presence of (**a**) 0.5, (**b**) 1.0, (**c**) 2.0, and (**d**) 3.0 μM Pb(II) ions concentrations.

We still face a challenge for a complete understanding of the quenching mechanism of Pep-CuNCs by Pb(II). Also, the mechanism could be attributed to the chelation of the Pb(II) by the function groups of the aspartic acid that presented in the pepsin structure; this brought the Pb(II) cations in the vicinity of the nanoclusters’ surface. The quenching mechanism was produced by the metal-complex production between the pepsin molecules on the surface of the nanoclusters and the studied metal ions Pb(II). However, this proposed mechanism is supported by the DLS data (Figure 6). The size of CuNCs did not considerably affect the presence of Pb(II) ions within different concentrations series involving 0.5, 1.0, 2.0, and 3.0 μM. No agglomeration was noticed for the studied samples. Therefore, we established that the luminescence quenching of the CuNCs was not evolved by the accumulation of Pep-CuNCs into larger particles.

### 3.4. Selectivity and Reversibility

**Figure 7:**
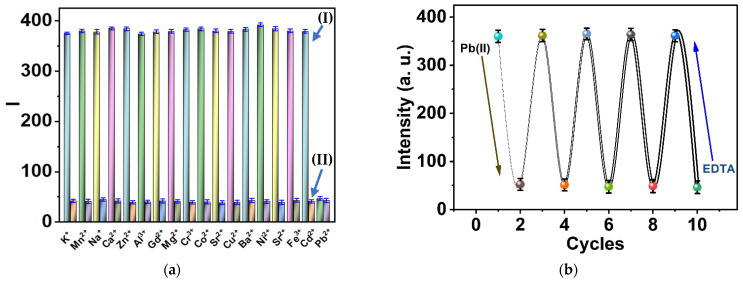
(**a**) Pep-CuNCs’ fluorescent intensities in the presence of (I) different metal ions and (II) different metal ions and Pb(II); (**b**)The recovery test based on EDTA solution; (*n* = 3).

To investigate the Pep-CuNCs selectivity, the impacts of diverse metal cations on the as-prepared Pep-CuNCs were examined under the same conditions in aqueous solution (Figure 7a). No substantial emission intensity change was detected in the presence of these studied cations, which confirms that CuNCs can successfully determine Pb(II) quantitively. Moreover, EDTA solution greatly enhanced CuNCs’ fluorescence in the Pep-CuNCs-Pb(II) system. Because Pb(II) cations react to the functional groups of EDTA molecule, therefore, Pb(II) was successfully liberated from the Pep-CuNCs surface, which restored the emission of CuNCs. Curiously, the recovery of Pep-CuNCs’ fluorescence was attained by approximately 97.8% percent of the initial emission in the presence of EDTA, as shown in Figure 7b. The fluorescence of CuNCs ddid not change as the number of cycles increased and reached similar fluorescence after four cycles. Moreover, the kinetic alterations in the cycles’ intensities produced significantly selective Pb(II) ions. Therefore, the recovery shown by the Pep-CuNCs method for Pb(II) detection was acceptable over four cycles.

### 3.5. Detection of Pb(II) Ions in Environmental Samples

The detection of Pb(II) metal ions using Pep-CuNCs in environmental samples (tape and mineral water samples) was examined. The tested water samples were used directly without any additional treatment. Moreover, certain Pb(II) metal ions concentrations in Milli-Q water were added to the real samples. Notably, the net results are based on the standard curve from the lab experiments. The concentrations of Pb(II) in tape and mineral water samples were successfully determined. The results are given in Table 1; the detection average recoveries are 96.20%, 98.90% and 99.10% for Pb(II) in tap water and 97.40%, 99.10% and 99.55% for Pb(II) in mineral water. The obtained results were compared with the measured data from ICP-MS instruments, proving that this sensing technique is promising for Pb(II) detection in environmental samples.

### 3.6. Cytotoxic Activity

**Figure 8:**
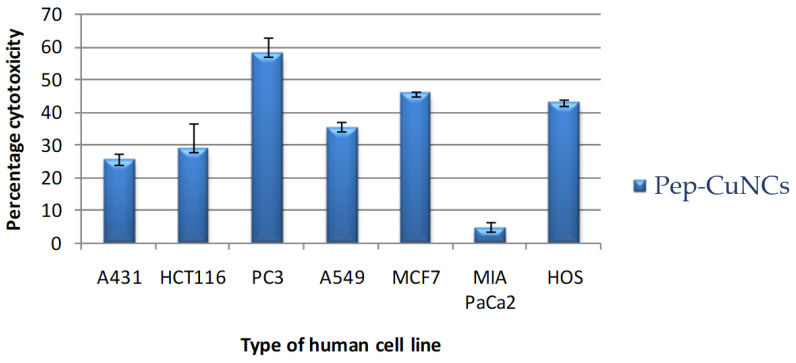
Cytotoxic activity of Pep-CuNCs at 100 µM.

The anticancer activity of the synthesized compound was tested on a series of cancer cell lines, namely, human epidermoid carcinoma (A431), colorectal carcinoma (HCT-116), prostate adenocarcinoma (PC3), lung adenocarcinoma (A-549), breast cancer adenocarcinoma (MCF-7), pancreatic carcinoma (PaCa2) and osteocarcinoma (HOS) cancer cells, using the MTT assay [30,31,32]. The results are shown in Table 1 and Table 2 and Figure 8, expressed in Table 2 as the cytotoxic effect of the samples at values of 100 µM and IC_50_ which reflect the effect of the synthesized particle on the cancer cells in the current investigation.

The results showed that the tested particle generally displayed its activity against the majority of cancer cell lines in a dose-dependent manner (Table 3). The tested compound Pep-CuNCs revealed a degree of selectivity against certain cancer cells (HOS, MCF7 and PC3) while the others tested were capable of only weak influence. It has been found that the HOS cancer cell was the most susceptible cell under all tested concentrations. The highest inhibition was observed for the PC3 at 100 µM, revealing 57.9 ± 4.8 as a percentage of inhibition. Furthermore, the cancer cells most susceptible to the action of the tested particle were identified as MCF7 and PC3 at 100 µM cells, while the least affected cancer cells tested were the HCT116 and PaCall cell lines. A significant difference can be observed in the displayed activity results for cells such as PC3 and PyCall.

Interestingly, minimal effects were observed for all the compounds tested on the RPE1 Human retinal pigment epithelial normal cell line at the studied concentrations (100, 50, 25 and 12.5 µM), which revealed interesting selectivity of activity against cancer cells without affecting normal cells.

## 4. Conclusions

This study firstly reports that Pep-CuNCs can be produced by a simple eco-friendly microwave-assisted approach, using pepsin enzyme as a capping and reducing agent. We synthesized an optical nanosensor based on CuNCs to sense Pb(II) ions. The considerable chelation attendance of the pepsin on CuNCs to Pb(II) metal ions led to quenching of the intensity of fluorescence in the nanoclusters, accompanying the quantitative detection of Pb(II). The applied Pep-CuNCs sensor displayed a high selectivity to Pb(II) ions in the presence of different types of metal ions. The reversibility of the sensor was examined in the presence of EDTA and results showed high recovery performance for four cycles. The proposed work was to effectively determine Pb(II) metal ions in real samples. The activity of Pep-CuNCs on various human tumor cell lines was examined and the results show that the Pep-CuNCs exhibited their activity against the cancer cells in a dose-dependent way in terms of the detected behavior against most utilized cancer cells lines. Finally, this method is suitable for precise quantitative determination of the hazardous metal ion in the study.

## Figures and Tables

**Table 1:** Sensing of Pb(II) in environmental samples based on Pep-CuNCs as a promising sensor (*n* = 3).

Samples	Added Pb(II) µM	ICP-MS µM	Found µM	RSD (%)	Recovery (%)
Tap water	0.50	0.513	0.481	1.35	96.20
	1.00	1.009	0.989	0.99	98.90
	2.00	2.007	1.982	0.92	99.10
Mineral water	0.50	0.511	0.487	1.29	97.40
	1.00	1.007	0.991	0.97	99.10
	2.00	2.005	1.991	0.96	99.55

**Table 2:** Activity of Pep-CuNCs on different human tumor cell lines.

Cell	Activity at 100 µM	IC_50_ (µM)
A431	25.1 ± 2.3	-
HCT116	28.9 ± 7.4	-
PC3	57.9 ± 4.8	-
A549	35.2 ± 1.6	-
MCF7	45.9 ± 0.3	104.4 ± 16, r^2^ = 0.94
PaCaII	4.6 ± 1.9	-
HOS	42.9 ± 0.9	-

**Table 3:** Activity of Pep-CuNCs on tumor cell lines.

Cell	100 µM	50 µM	25 µM	12.5 µM
HOS	42.9 ± 0.9	50.9 ± 1.3	40.8 ± 2.4	17.8 ± 1.6
MCF7	45.9 ± 0.3	29.2 ± 0.5	3.6 ± 2.2	0.0 ± 0.0
PC3	57.9 ± 4.8	0.0 ± 0.0	0.0 ± 0.0	0.0 ± 0.0
A549	35.2 ± 1.6	0.0 ± 0.0	0.0 ± 0.0	0.0 ± 0.0
A431	25.1 ± 2.3	0.0 ± 0.0	0.0 ± 0.0	21.4 ± 2.1
HCT116	28.9 ± 7.4	0.0 ± 0.0	0.0 ± 0.0	0.0 ± 0.0
PaCaII	4.6 ± 1.9	0.0 ± 0.0	0.0 ± 0.0	0.0 ± 0.0

## Data Availability

Not applicable.
